# Causes of infertility in view of Iranian traditional medicine: A review

**Published:** 2017-04-10

**Authors:** Seyed Kazem Kazemeini, Majid Emtiazy, Fatemeh Owlia, Parisa Khani

**Affiliations:** 1 *Department of Traditional Medicine, Faculty of Iranian Traditional Medicine, Shahid Sadoughi University of Medical Sciences, Ardakan, Iran.*; 2 *Department of Oral Medicine, Dental Faculty, Shahid Sadoughi University of Medical Sciences, Yazd, Iran.*; 3 *Research and Clinical Center for Infertility, Yazd Reproductive Sciences Institute, Shahid Sadoughi University of Medical Sciences, Yazd, Iran.*

**Keywords:** Infertility, Iranian traditional medicine, Male infertility, Female infertility, Mizaj

## Abstract

Infertility is one of the most important reproductive health concerns in the conventional medicine. Iranian traditional medicine presents different viewpoints in this regard which they could be of benefit and a good guide for the society of medicine. This study sought to provide the comprehensive investigation on the causes of infertility according to Iranian traditional medicine for understanding of old sages' ideas and categorizing of the causes of infertility. In this narrative review, we searched causes of infertility in traditional medicine books and available articles in this field. Iranian traditional physicians have investigated the causes of infertility in couples and attributed them to male and female causes. They have divided the main causes of infertility in both sexes into structural and functional abnormalities, that both traditional medicine and conventional medicine have a lot of participations, but the traditional medicine believes holistic approach in the treatment of diseases and the involvement of all parts of the body particularly specialty board members (heart, liver, brain, ovary, and testicles) in the proper conduct activities in different parts of the body such as reproduction system. There is also special attention to temperament Mizaj disorders. Given the numerous commonalities existing between traditional and conventional medicine in categorizing the causes of infertility, Iranian traditional medicine methods can be applied as a complementary solution in infertility. It could be also subject to further research and investigation due to its opposition to modern medicine in some regards.

## Introduction

Infertility is defined as couples' inability in childbearing despite one year beginning of regular and unprotected intercourse (1). This period for women over 35, however, has been considered six months (2). The inability of couples in childbearing presents serious social and economic problems which are costly and annoying for families involved. Accordingly, it has been in the spotlight in the world of medicine for a long time. Despite remarkable advances in diagnostic techniques and treatment of infertility, the prevalence of infertility has been on the rise in recent years. According to the World Health Organization report, almost one out of six couples face infertility problem and a total of 80 million people dealing with fertility failure worldwide (3). The prevalence of infertility in different countries is estimated from 3.5-22% (4). The frequency of infertility in Iran, has been reported 10.6%. Nearly, 25% of Iranian couples have experienced infertility during their married life (5). In an investigation conducted on 15000 men and women in England between 2010 and 2012, it was mentioned that about 12.5% of women and 10.1% of men were afflicted with primary infertility (6).

Studies indicated that 30% of infertility is related to male factors, 45% are due to female factors, and 25% have no known cause (7). The growing tendency in couples to postpone child bearing and changes in sexual behavior and lifestyle could explain the upward trend in infertility (8). However, a considerable number of unexplained infertilities, in spite recent advances in diagnosis and treatment of infertility, could represent unknown causes of this disease that can be addressed through experiences gained in Iranian traditional medicine and old sages. Physicians in ancient Iran have paid particular attention to infertility and have allocated separate parts of their books to it, methods of its identification, and treatment. Infertility has been called [aqr] (the lack of carrying a pregnancy), [osrolhaball] (difficulty in pregnancy), [aqimi](barenness), and [oqm] (not getting pregnant) in Iranian traditional medicine (9, 10). 

This study conducted the comprehensive investigation on the causes of infertility according to Iranian traditional medicine for an understanding of old sages' ideas and categorizing of the causes of infertility to open a window for future progress to find the new causes of infertility.

## Materials and methods

In this narrative review, the following keywords such as “aqr”, “osrolhaball”, “aqimi”, and “oqm” being searched in books named Al Qanon Fi Al-tibb, Zakhireh Kharazmshahi, Tibb Akbari Exir Azam, Moalejate Aghili, Kholaseh Al-Hekmat. Materials relevant to the causes of infertility were extracted from the above-mentioned sources. In the next phase, Google scholar, Medline, and PubMed were searched for recent articles (such as review articles, systematic review, clinical trials, and demographic articles) published from 2005 to 2016 by keywords including Iranian traditional medicine, Persian medicine, infertile and infertility.

## Results

In the books of Iranian traditional medicine, infertility was defined as; "a woman who does not get pregnant, or gets so hard and its causes can be blamed on man or woman or both" (11). According to ancient Iranian medicine, in confronting an infertile couple, at first, male problems should be evaluated (12). Scientists talked about the production and secretion of semen and attributed the embryo formation to the combination of semen from man and woman since many years ago. They assigned the causes of infertility to the content, secretion pathway, and the quantity of semen problem in both sexes (13). In Iranian traditional medicine, different methods have been used to detect which one of the spouses (man or woman) is blamed for infertility, for instance, semen from both was poured on water separately, each one floats it considered as infertile. 

Another method used for evaluating the cause of female infertility, was to place garlic in a piece of cloth and then in the woman's vagina at night and during sleep. If she felt the smell or the taste of it in her mouth the next morning, it was a sign of lack of obstruction in the woman's genital track of semen passing (12). 

In general, books of Iranian traditional medicine have called the following as the major causes of infertility: 1) Abnormality in quality and quantity of semen in man, woman or both, 2) Uterine or female reproductive system abnormalities, 3) Abnormality in male reproductive system and semen production or function, 4) Medical conditions in other organs are related to the reproductive system of both sexes, 5) The occurrence of some mistakes in sexual intercourse (11, 14, 15) (Table I).

**Causes of male infertility**

According to Iranian traditional medicine, the male reproductive system includes the testicles as responsible for producing semen, [qazib] (penis), and the tubes leading sperm away from the testes to the penis (16). Qazib (penis) is comprised of the nerves, arteries, veins, and muscles which the urethra and seminal duct are located between them (17). The ancient philosophers believed that main substance of semen originates from the brain, passes through the two vessels behind the ear, and finally reaches the testicles. They believed that by cutting off the two vessels, male infertility generally disappears (12 ,18).

***1. Anatomical and structural abnormalities***

A) Penis abnormality which is attached to following causes: the presence of an arch, the problem in penis size (too big or small penis), semen outlet deviation from the midline, weakness of the penis which impairs the entry of semen into the uterus (19).

B) Injury to penis nerves or its blood vessels, which impairs the formation and transport of semen.

C) Testicles’ damage following trauma, which can disrupt the semen production. 

D) Traditional scientists believed that male obesity poses an obstacle for transportation of semen to the uterus.

E) The presence of the swelling or mass in the testicles leads to decreased formation or transfer of the semen.

F) [Davali] (which is known as varicose in modern medicine) (12, 14, 20).

***2. Semen abnormalities***

A) Low semen volume due to reducing food intake, being underweight, drug abuse, or rigorous sports.

B) Low semen quality which is attributed to the change in its temperament Mizaj bases (21). In Iranian traditional medicine, Mizaj is a quality known as comprised of the four elements (water, air, fire, and soil). Whenever its balance is disrupted, disease would be happened (22). Semen in Iranian traditional medicine has been defined as a steadily thick liquid, of brightly white color, and mild temperature which smells like the Palm-tree blossoms and is formed in testicles. They believed that if it gets out of its base Mizaj, it will pose problems in semen quality.

C) Excessive consumption of some materials likes camphor or opium.

D) The robbing of (šo[w]karān) (which is a poisonous plant) on the scrotum (12, 14, 18).

***3. Other organs disorders***

Iranian traditional medicine philosophers looked at the human body as a comprehensive unit and a system that its parts working cooperatively. They believed that the presence of a problem in one organ particularly the major parts (heart, brain, liver, testicles, or ovaries) and gastrointestinal organs such as stomach could have an effect on the other parts. Therefore, any problems in theses organs were considered as causes of semen abnormality (23, 24).

***Psychological problems ***such as fear, anger, and sadness.

***5. Sexual disorders***

The abandoning of sexual intercourse for a long time which has been mentioned as a factor in the reduction of the semen (11, 12, 20)

***6. Unknown***

Infertility with unknown causes or real infertility; if traditional medicine philosophers couldn't attach infertility to any reasons in their investigations, they would name it "real infertility" (20).

**Causes of female infertility**

Woman's reproductive system, according to Iranian traditional medicine, comprised of uterus, ['onoqelrahem] (vagina), [beyze al-rahem] (ovaries), and ['o[w]'iye] (fallopian tubes). In Iranian traditional medicine, the embryonic origin of the uterus was considered to be a nerve and classified into two parts: the inner part with a lot of vessels and stomach-like rugae and the outer part which functions as a sheath and covering and is linked to its surroundings through some ligaments. ['onoqelrahem] (Vagina in modern medicine) listed a muscular organ like cartilage that its junction to uterus was named [fam] (cervix in modern medicine). Ovaries have been considered as counterparts to men's testicles but they are flatter, smaller, and firmer. It was considered as the place in which woman's semen is formed and junctioned with uterus through [ooeeya] (equivalent for Fallopian tubes in conventional medicine) (18, 20, 25).

In Iranian traditional medicine, the causes of infertility in women include the following: 

***1. Obstructive causes***

Traditional physicians were of the belief that whatever causes disrupt in the man or woman's semen transferring into the uterus can be caused infertility. Some of its reasons are mentioned below: 

A) The obstruction in the passage of the semen through the uterus (the female reproductive tract) caused due to internal or external factors. Internal factors were considered: wart, hemorrhoid [bavāsir] or deviation of the uterus (26). The deviation of the uterus causes the inappropriate placing of the uterus along the vagina therefore not reaching of man's semen to the uterus. Of its symptoms was mentioned the pain during intercourse which was detectable through the examination by an expert. Also, hard swellings of the uterus like tumors and the straining or the toughening of uterus ligaments in one side been mentioned as its causes (11,13, 27). 

The external factors referred to trauma to the cervix or uterus that could lead to scarring or adhesions or foreign bodies in the vagina (12). Traditional physicians said the accumulation of the wind in the uterus was called the flatulence of the uterus. This problem is said to be responsible for the lack of implantation of the semen. The other symptom is deterioration of exiting wind from uterus during intercourse after eating flatulent food, , and the feeling of flatulence between the navel and the genital organ (11, 12).

B) Obesity especially in people with big abdomen through excessive pressure on the uterus, fallopian tubes, and vagina causes the obstruction of their track which has been mentioned the cause of infertility (10).

C) The presence of too much fat on uterine wall which through the prevention of accepting the implantation of the sperm is among the causes of infertility.

D) Protrusion of the uterus.

E) The weakness of the cervix.

F) [salābat] (The toughness of the uterus).

***2. Dystemperament (su’ mizaj) in the female reproductive system:***

Traditional physicians believed that dystemperament in the uterus could cause structural disorders, corruption of semen temperament, and the inability of the uterus to embryo holding. They categorized it into the following types:

A) Changes in Mizaj of the uterus towards burudat (coldness): which causes fallopian tube stenosis or obstruction, the problem in the woman's semen movement or slow that, and resulting infertility. Also, traditional physicians believed that a cold uterus reduces the fertilization ability because the moderate temperature is necessary for normal function of body organs. In Iranian traditional sources, the following symptoms have been mentioned for change in Mizaj of the uterus towards coldness: Oligomenorrhea (menstrual bleeding occurring more than 35 days apart and witch remains constant at that frequency), decrease viscosity of menstrual blood, prolonged menstrual periods, less pubic hairs, and the cold abdomen skin in the place of the uterus. 

B) Changes in Mizaj of the uterus towards Harr (Hot) which causes corruption of semen temperament. As mentioned above, the moderate temperature is required for the appropriate activity of the uterus and reproductive system. However, high heat could pose problems. The following symptoms were mentioned for Harr (Hot) Mizaj of the uterus: dark reddish black menstrual blood, increase viscosity and temperature of menstrual blood, hypomenorrhea (when the menstrual bleeding is unduly scan and lasts for less than 2 days) excessive pubic hairs, and the warm abdomen skin in the place of the uterus.

C) Changes in mizaj of the uterus towards dryness: This change in the mizaj was also mentioned as one of the reasons for corruption of semen temperament. The excessive dryness of the uterus has been considered as a factor for reduction sperm motility and consequently the fertilization. They believed that even the fertilization occurs, the possibility of appropriate implantation and embryo survival is low. For these women in traditional medicine following symptoms were mentioned: Less and dark menstrual blood, less vaginal discharge (Vaginal Dryness), and body slimming. 

D) Changes in Mizaj of the uterus towards Ratb (Wet): Iranian traditional literatures have considered its effect on infertility as the flaccidity of the uterine wall and the weakness in uterus' attraction and holding power. Traditional physicians have considered the extreme wetness of the uterus as a factor for the corruption of semen. They have mentioned due to the poor uterine environment, even the fertilization occurs the appropriate implantation and embryo survival is not possible. Hence, they have taken this change in the temperament (su’ mizaj) as one of the reasons for miscarriage. Of the symptoms of this su’ mizaj is increased the amount of vaginal discharge, lack of cervical dryness after the end of the menstrual cycle, and puffiness of the face and body (11, 14 and 15).

E) The material type of the complex dystemperament: From the Iranian traditional medicine point of view, the body is comprised of the four humors (Akhlat): Dam (blood), Balgham (phlegm), Safra (yellow bile), and Sauda (black bile). (28) The pouring of Balgham, Safra, and Sauda on the uterus is considered as another reason for infertility in traditional medicine. It poses problems for the uterus which is called material dystemperament. Uterine secretions of these women are back, yellow, and white, respectively based on the type of their humors (Akhlat) (11).

***3. The absence of menstrual bleeding (Amenorrhea)***

In Iranian traditional medicine, the menstrual bleeding has been mentioned as the source of nutrition for the fetus. In its absence, the continuation of life is disrupted. (13) 

***4. Thinness***

In Iranian traditional medicine, thinness is known to be an obstacle for the producing of high-quality semen. Even if the fertilization occurs, their body does not provide good food for the fetus and the survival of the fetus is not possible (12). The flatulence of the uterus is kind of complex dystemperament that can be cause of female infertility (17). 

***5. Psychological problems***

Have been mentioned as another cause for infertility with women being affected more (29).

***6. Other organs disorders***

As mentioned earlier, Iranian traditional medicine considered the human body as a comprehensive unit and problem in each its organs particularly the major parts (heart, brain, liver, testicles, or ovaries) and gastrointestinal system such as stomach were considered as causes of infertility (13).

***7. Intercourse disorders***

A) Women's getting up or rigorously moving after sexual intercourse.

B) Recurrent sexual intercourses which prevent the staying of sperm in the uterus.

***8. Other reasons***

Which have been listed for women's infertility in Iranian traditional medicine:

- Taking a bath a lot could lead to infertility, because of uterus laxity.
- Uterus incapable.

***9. Unknown ***(13, 20, 26).

## Discussion

As mentioned in previous sections, Iranian traditional philosophers were familiar with the anatomy of male and female reproductive systems which has a lot in common with the conventional medicine. However, they are named differently in the latter. In addition, there is special attention to infertility issue in Iranian traditional medicine in both sexes as implicated in the disease and its causes and treatment have mentioned in detail.

In conventional medicine, causes of male infertility classified into pre-testicular, testicular, and post-testicular causes. Pre- testicular causes e.g. hormonal disorders (hypothalamus, pituitary, thyroid disorder), and systemic disorders (hepatic, renal, etc.) (30). As previously mentioned, the human body is looked upon as a comprehensive unit and problem in each its organs will have an effect on the other parts (31). Conventional medicine also affects some organs such as the brain, referred to male infertility that maybe in the future the role of other organs such as the heart and stomach is realized. Additionally, philosophers following traditional medicine assumed the brain to have an important role in producing the main substance of the semen. In this regard, conventional medicine considered disorders of the hypothalamic-pituitary-gonadal axis which are located in the skull and in the proximity of the brain effective on male infertility. Moreover, thyroid disorders due to dystemperament towards burudat (coldness) or Harr (Hot), which in traditional medicine have been mentioned as causes of infertility, may affect fertility (17).

In conventional medicine, testicular causes are known as one of the major causes of male infertility. Testicles produce sperm (during spermatogenesis) and any problem in this organ including infection, trauma, varicocele, contact with chemicals or radiation, cryptorchidism, and chromosomal anomalies can be a cause of infertility (32). [Davali] (varicose) and [Nahan khayegi] (cryptorchidism) also been mentioned in traditional medical sources. While chromosomal anomalies have not been mentioned in traditional medicine (12).

Post-testicular causes of infertility in conventional medicine include seminiferous tubules obstruction, infection or injury as well as damage to the sympathetic nervous system, disorders of sperm motility and maturation, and problems related to the penis and physical disability (33). These causes mentioned as obstuctive in semen pathway in traditional medicine (12).

Environmental factors like heat or infection which have been taken as causes for infertility in conventional medicine. According to Iranian traditional medicine, these factors cause infertility through dystemperament (su’ mizaj) in men and reproductive organs (34). As regards 30% of male infertility factors is yet unknown in conventional medicine, the investigation of other possible causes seems necessary. Among them, the role of the stomach, nutrition, the appropriate digestion of food, mental and psychological issues, the observation of necessary for good health (weather, foods and drinks, physical activity, sleep, retention and expulsion, mental and psychological issues) as well as the role of other organs and dystemperament. 

Of the most important causes of female infertility in conventional medicine is the ovarian dysfunction by damaging follicles and their oocytes which have been named as the disorders in the quantity and quality of the semen. The dystemperamnet of the semen which is the most important quality disorder in women's semen is assumed to be one of the major causes of female infertility. Conventional medicine takes brain system responsible for the process of ovulation which has been named the weakness of [Damaq]. 

As mentioned earlier, as a principle in traditional medicine, different parts of the body work cooperatively as a unified system. Other organs like brain, liver, heart, and stomach play a role in the formation of semen in women. Conventional medicine has paid insufficient attention to this fact. In addition, nutrition as a fundamental principle in the process of all bodily activities including reproduction has been taken into consideration in traditional medicine, unlike conventional medicine (13, 35). Structural disorders of the uterus like tumors are other causes of female infertility in conventional medicine. This has been variously named in traditional medicine such as [sodde], the additional mass in the uterus, today it was named fibroma, the hemorrhoid of the uterus, uterus stiffness, the swelling of the uterus, the tough swelling at of the cervix, the uterus deviation, and obesity. Previously, the methods for investigating the obstruction of the fallopian tubes in traditional medicine were mentioned (the garlic test in vagina) (13, 36).

However, some causes the uterus flatulence have been referred to Iranian traditional medicine, that conventional medicine does not mention (13). Both conventional and traditional medicine has assumed a role for mental and psychological problems among the causes of infertility. This has been referred to as ['a'rāz] in traditional medicine (29, 37).

## Conclusion

Given the numerous commonalities existing between traditional and conventional medicine in categorizing the causes of infertility, Iranian traditional medicine methods could be applied as a complementary solution in infertility. It could be also subject to further research and investigation due to its opposition to conventional medicine in some regards.

## Conflict of interest

There is no conflict of interest for authors. 
